# Mouse Genome-Wide Association Mapping Needs Linkage Analysis to Avoid False-Positive Loci

**DOI:** 10.1371/journal.pgen.1000331

**Published:** 2009-01-09

**Authors:** Giacomo Manenti, Antonella Galvan, Angela Pettinicchio, Gaia Trincucci, Elena Spada, Anna Zolin, Silvano Milani, Anna Gonzalez-Neira, Tommaso A. Dragani

**Affiliations:** 1 Department of Experimental Oncology and Laboratories, Fondazione IRCCS Istituto Nazionale Tumori, Milan, Italy; 2 Istituto di Statistica Medica e Biometria “GA Maccacaro”, Università di Milano, Milan, Italy; 3 Genotyping Unit (CeGen), CNIO, Madrid, Spain; The Jackson Laboratory, United States of America

## Abstract

We carried out genome-wide association (GWA) studies in inbred mouse strains characterized for their lung tumor susceptibility phenotypes (spontaneous or urethane-induced) with panels of 12,959 (13K) or 138,793 (140K) single-nucleotide polymorphisms (SNPs). Above the statistical thresholds, we detected only SNP rs3681853 on Chromosome 5, two SNPs in the pulmonary adenoma susceptibility 1 (*Pas1*) locus, and SNP rs4174648 on Chromosome 16 for spontaneous tumor incidence, urethane-induced tumor incidence, and urethane-induced tumor multiplicity, respectively, with the 13K SNP panel, but only the *Pas1* locus with the 140K SNP panel. Haplotype analysis carried out in the latter panel detected four additional loci. Loci reported in previous GWA studies failed to replicate. Genome-wide genetic linkage analysis in urethane-treated (BALB/c×C3H/He)F2, (BALB/c×SWR/J)F2, and (A/J×C3H/He)F2 mice showed that *Pas1*, but none of the other loci detected previously or herein by GWA, had a significant effect. The *Lasc1* gene, identified by GWA as a functional element (Nat. Genet., 38:888–95, 2006), showed no genetic effects in the two independent intercross mouse populations containing both alleles, nor was it expressed in mouse normal lung or lung tumors. Our results indicate that GWA studies in mouse inbred strains can suffer a high rate of false-positive results and that such an approach should be used in conjunction with classical linkage mapping in genetic crosses.

## Author Summary

Genome-wide mapping is now popular in both humans and experimental animals, but results of these studies are not validated by independent approaches. We conducted a genome-wide mapping analysis of lung cancer phenotypes in mouse strains and compared our results with those in three previous studies. We found that most of the loci identified in the earlier studies do not replicate. When we combined genome-wide association study with genetic linkage analysis, representing the gold standard of causal inference for allelic effects, the *Pas1* locus detected in only one of the three previous genome-wide studies did replicate in genetic crosses, whereas the reportedly functional *Lasc1* D102E polymorphism lacked allelic effects in two independent crosses. Our study supports the notion that association mapping in the population of inbred mouse strains is characterized by a high false-positive rate and that such a method must be carried out in conjunction with linkage analysis to detect relevant loci. These results point to the need for independent confirmations in population-based studies.

## Introduction

Association studies are based on linkage disequilibrium (LD) between genetic markers and a disease locus affecting a particular phenotype [1],[2]. Such studies may allow the fine mapping of loci affecting monogenic diseases, as well as loci affecting complex diseases if the relevant alleles are common in the general population under investigation. This approach can be quite powerful when disease chromosomes are descended from a single founder mutation and the markers analyzed are tightly linked to the disease locus. In these cases, the LD approach has proven successful not only in humans for the fine mapping of rare diseases in isolated populations, but also in experimental animals. Indeed, our LD analysis in mouse inbred strains served in refining the mapping region of *Pas1*, the major locus affecting susceptibility to mouse lung tumorigenesis [3]. Subsequently, we found that the *Pas1* locus consists of a conserved haplotype spanning ∼400 kb and including 6 genes, whose polymorphisms define a susceptibility allele that is frequent in laboratory mouse inbred strains and apparently derived from an ancestral progenitor [4]. More recently, we used LD analysis to show that the *Pas1* locus affects not only carcinogen-induced lung tumor multiplicity, but also spontaneous lung tumor incidence [5].

The availability of single nucleotide polymorphism (SNP) data for many inbred strains has led to the proposal of *in silico* genome-wide mapping of mouse quantitative trait loci (QTLs) [6]. In three genome-wide association (GWA) studies aimed at detecting lung tumor modifier loci, 5 loci (named *SLT* loci) affecting incidence of spontaneous lung tumors [7], 4 loci (named *Clas* loci) affecting incidence of urethane-induced lung tumors [8], and 5 loci affecting incidence of N-nitroso-N-ethylurea-induced lung adenomas or adenocarcinomas [9] were identified. Each of these three reports detected a unique, non-overlapping set of loci, despite analysis of similar populations of mouse strains and highly correlated tumor phenotypes, i.e., spontaneous, urethane-, or N-nitroso-N-ethylurea-induced lung tumor incidences [5].

The identification of authentic lung tumor modifier loci in the population of mouse inbred strains is fundamental to revealing the genetic elements that control tumor susceptibility in this mammalian model. To assess the reproducibility and functional relevance of GWA results, we carried out this analysis using a larger number of mouse strains and more phenotypes describing the lung tumor susceptibility trait than in previous studies and compared the results with those obtained from standard genetic linkage analyses in intercross populations.

## Results

### GWAs Detect Putative Lung Tumor Modifier Loci

**Figure 1:**
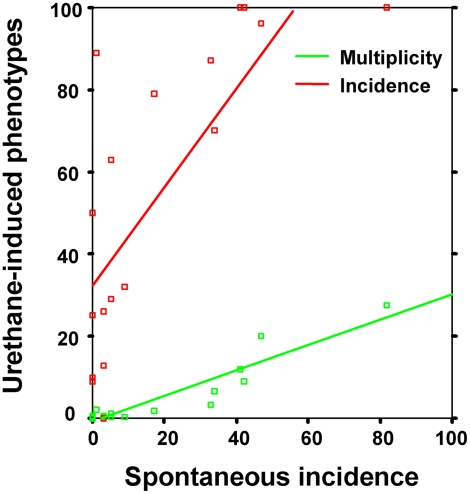
Spontaneous lung tumor incidence correlates with both urethane-induced lung tumor multiplicity (green) and incidence (red) in mouse inbred strains. Incidence is given as mean percentages, whereas multiplicity is mean number of tumors/mouse. See Table 1 for phenotype values.

Strain susceptibility to lung tumorigenesis can be described using different phenotypes, such as tumor incidence, tumor multiplicity, and tumor volume. Mouse inbred strains show a wide range of lung tumor phenotypes, with mean spontaneous tumor incidence ranging from 0 to 82%, mean urethane-induced lung tumor incidence from 0 to 100%, and mean urethane-induced lung tumor multiplicity ranging from 0 to 28.3 tumors/mouse (Table 1). A highly significant correlation was found between the spontaneous tumor incidence and urethane-induced tumor multiplicity phenotypes (r = 0.94, −log P = 8.9), whereas correlation between the spontaneous tumor incidence and urethane-induced tumor incidence phenotypes was weaker (r = 0.76, −log P = 3.8) (Figure 1).

**Table 1:** Lung tumor phenotypes (spontaneous and urethane-induced incidence and urethane-induced tumor multiplicity) of mouse inbred strains used in this study.

Strain	Spontaneous Incidence (mean %) 1	Urethane-induced Incidence (mean %) 2	Urethane-induced Multiplicity (no. of tumors/mouse) 3	SNP Database 4
129X1/SvJ	1	89	2.1	W, B
A/J	82	100	27.5	W, B
AKR/J	0	9	0.1	W, B
BALB/c	33	87	3.3	W, B
C3H/HeJ	9	7	0.2	W, B
C57BL/10J	3	26	0.3	W
C57BL/6J	3	13	0.5	W, B
C57BR/cdJ	3	0	0	W, B
C57L/J	0	10	0.1	W, B
C58/J			0.5	W, B
CAST/Ei	0			W, B
CBA/J	17	79	1.7	W, B
CE/J	0			W, B
DBA/1J	3			W, B
DBA/2J	0	25	0.4	W, B
FVB/NJ	36			W, B
LP/J	5	63	1.1	W, B
MA/MyJ	42	100	8.9	W, B
NGP			28.3	
NZB/BlNJ	0	25	0.3	W, B
NZW/LacJ	24			W, B
O20	41	100	12.1	B
P/J	3			W
PL/J		67	2	W, B
RF/J	26			W
RIIIS/J	34	70	6.7	W, B
SJL/J	5	29	0.3	W, B
SM/J	0	50	0.5	W, B
SPRET/EiJ	0		0.5	W, B
ST/bJ		86	3.2	W, B
STS/A	24			W
SWR/J	47	96	20.1	W, B

1 Mean incidence of spontaneous lung tumors; data from [5], except for CAST/Ei, NZW/LacJ, SM/J, and SPRET/EiJ, which derived from [7].

2 Mean incidence of urethane-induced lung tumors; data from [5] or [22].

3 Mean lung tumor multiplicity after urethane treatment; data from [5], except for SPRET/EiJ, which derived from [23].

4 W, WTCHG; B, BROAD.

GWA using the WTCHG 13K SNP panel was carried out in 20 to 27 strains for which phenotype-genotype data were available (Table 1). For any of the lung tumor phenotypes, the Bonferroni's statistical threshold (α = 0.1 significance level) accounting for the number of statistical tests (i.e., 12,959) would result in a −log P value = 5.1. GWA analyses revealed that only rs3681853 on Chromosome 5 reached this statistical threshold for spontaneous tumor incidence, whereas no SNPs were associated with urethane-induced tumor incidence and only one SNP (rs4174648) on Chromosome 16 was associated with urethane-induced tumor multiplicity. Surprisingly, the known *Pas1* locus was not detected. Using the suggestive thresholds obtained by permutation tests (α = 0.10, Table 2), which also account for the correlation structure of the data, we detected only SNP rs3681853 on Chromosome 5, (−log P = 5.3, spontaneous incidence, *SLT6*), SNPs rs13459098 and rs13479086 in the *Pas1* region (both SNPs at −log P = 4.7, urethane-induced tumor incidence, *Clas1*) and SNP rs4174648 on Chromosome 16 (−log P = 6.5, urethane-induced tumor multiplicity, *Clas5*) (Table 3). When *Mus spretus* was excluded from the analysis, rs3681853 did not reach any statistical thresholds and the other results remained the same.

**Table 2:** Threshold probabilities (expressed as −log P) corresponding to experiment-wide type I risk errors α = 0.05 and α = 0.10.

	Lung tumor phenotype	WTCHG (12,959 SNP)		BROAD (138,793 SNP)	
		α = 0.05	α = 0.10	α = 0.05	α = 0.10
Permutation test	Spontaneous incidence	5.6	5.2	7.8	7.1
	Urethane-induced incidence	5.1	4.7	7.0	6.5
	Urethane-induced multiplicity	6.8	6.1	6.9	6.8
t-test (with Bonferroni's adjustment)		5.4	5.1	6.4	6.1

**Table 3:** Putative lung tumor modifier loci identified by previous genome-wide association studies or by the present study using the WTCHG SNP panel.

Locus a	SNP b	Chromosome	Position (Mb) c	Spontaneous incidence d	Urethane-induced incidence d	Urethane-induced multiplicity d
*SLT1*	rs29883445	6	84.19	2.2	0.2	0.7
*SLT2*	rs31152907	7	10.75	1.5	0.5	0.8
*SLT3*	rs13480027	8	126.47	1.0	0.9	1.4
*SLT4*	rs13483600	19	34.75	3.7	1.0	3.6
*SLT5*	rs3667513	X	139.92	3.1	1.6	2.1
*SLT6*	rs3681853	5	150.21	**5.3**	0.8	3.9
*Clas1 (Pas1)*	rs13459098	6	145.12	3.8	**4.7**	3.8
*Clas2 (Lasc1)*	rs32396036 (D102E)	4	30.36	1.8	2.4	1.9
*Clas3*	rs3690198	13	60.82	0.6	2.6	2.5
*Clas4*	rs13482741	15	100.95	0.5	2.4	2.5
*Clas5*	rs4174648	16	35.59	2.8	1.5	**6.5**

a *SLT1* to *SLT5* loci, reported to affect spontaneous lung tumor incidence in 13 strains [7]; *Clas1* to *Clas4* loci, reported to affect lung tumor multiplicity in 21 strains [8]. Additional *SLT* and *Clas* loci derive from the present study; for each locus region (1 Mb size), the SNP showing the best statistical association is shown.

b Selected SNPs mapping in the reported *SLT1* to *SLT5* or *Clas1* to *Clas4* regions were genotyped in all strains for which phenotypes were available (Table 1); the SNPs showing the highest association with the phenotype are shown.

c Position (in Mb) based on Ensembl release 49.

d The association between each SNP and lung tumor phenotypes (expressed as log+1 of phenotype value) was tested by t-test. Minus log P values reported in bold type indicate the associations above the statistical threshold obtained by permutation (at α = 0.10 significance level).

Due to the low sensitivity of detection of putative loci based on statistical thresholds, as demonstrated by the significant association of the known *Pas1* locus with only one of three lung tumor phenotypes, we also examined putative loci whose associations were below the statistical thresholds. For example, at −log P≥4, 10 and 11 SNPs showed significant association with spontaneous incidence (*SLT* loci) and urethane-induced multiplicity (*Clas* loci), respectively, of lung tumors. No other SNPs above −log P = 4, except the two *Pas1*-associated SNPs at −log P = 4.7, were detected for the phenotype “incidence of urethane-induced lung tumors.” By attributing a locus definition to chromosomal regions spanning less than 1 Mb in length and containing one or more SNPs associated with lung tumor phenotypes, these associations identified 8 new *SLT* loci that included *Pas1*, the previous *Clas1* (*Pas1*), and 5 new *Clas* loci (not shown).

We then replicated the GWA using the BROAD SNP panel, which provided a higher SNP density (140K) but a lower number of strains (i.e., 20 to 23) (Table 1). To reduce the risk of false-positives due to the inclusion of genetically distant strains, we excluded the *Mus spretus* strain (SPRET/EiJ). Above the suggestive thresholds (α = 0.10, Table 2), we detected only SNPs rs30118733 and rs30752783 (−log P = 7.5 and 6.9, respectively, urethane-induced incidence), both of which were located in the *Pas1* region. *SLT1* to *SLT6* and *Clas2* to *Clas5* were not confirmed by analysis using the BROAD SNP panel, although *SLT6* and *Clas5* were detected by the WTCHG SNP panel (Table 3).

**Figure 2:**
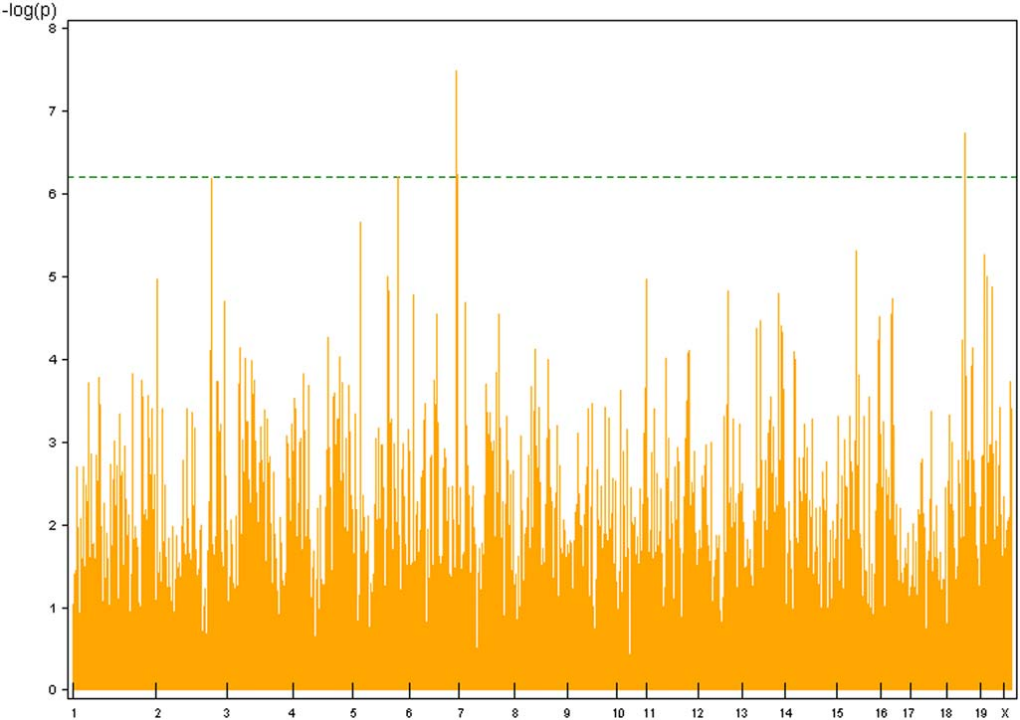
Genome wide scans for haplotype association with urethane-induced lung tumor multiplicity in mouse inbred strains using F-test for window size of 3 SNPs against the marker map plot. Threshold (in dotted green line) p value (α = 0.10) was calculated according to Bonferroni's criterion.

Finally, our haplotype-based GWA analysis using the BROAD dataset and a three-SNP sliding window revealed haplotype-associated lung tumor (*Halt*) loci (Table 4, Figure 2). For spontaneous lung tumor incidence, no haplotype reached statistical threshold (α = 0.10, Table 2), whereas for urethane-induced lung tumor incidence, two associated haplotypes were detected: *Halt1* in the *Pas1* region and *Halt2* on Chromosome 14 (Table 4). Associated to the urethane-induced tumor multiplicity phenotype, we detected five statistically significant haplotypes (*Halt3*- *Halt7*), two of which mapped in the *Pas1* locus (*Halt5*) or in its flanking region (*Halt6*) (Table 4).

**Table 4:** Haplotype-associated lung tumor modifier (*Halt*) loci identified by haplotype analysis, using the 140K BROAD SNP panel.

Locus	Markers	Chromosome	Position (Mb) a	Global -log P b	Lung tumor phenotype c	Intercross d
*Halt1 (Pas1)*	rs30907104–rs30843330–rs30118733	6	144.90	6.4	UI	AHF2, CHF2
*Halt2*	rs31236017–rs31387917–rs31237574	14	100.68	6.7	UI	-
*Halt3*	rs33459720–rs33172833–rs33360453	2	147.65	6.2	UM	AHF2, CWF2
*Halt4*	rs29683518–rs32191266–rs29505322	5	121.63	6.2	UM	AHF2, CHF2
*Halt5 (Pas1)*	rs30913614–rs30514198–rs29924904	6	145.13	7.5	UM	AHF2, CHF2
*Halt6*	rs3694732–rs30366423–rs52246948	6	147.51	6.2	UM	AHF2, CHF2
*Halt7*	rs29724114–rs30023351–rs29867331	18	59.45	6.7	UM	AHF2, CHF2, CWF2

a Position of the central SNP, in mega bases (Mb) based on NCBI m37 mouse assembly.

b Global -log P is the minus logarithm of the p-value for the haplotype sliding window (window size: 3-SNPs).

c UI, urethane-induced lung tumor incidence, UM, urethane-induced lung tumor multiplicity.

d At each *Halt* locus, the intercrosses whose parental strains carry different alleles, and that have herein been analyzed, are indicated. AHF2, (A/J×C3H/He)F2; CHF2, (BALB/c×C3H/He)F2; CWF2, (BALB/c×SWR/J)F2.

### Results of GWA Studies Fail to Replicate

A previous GWA study in 13 inbred strains detected 5 loci, named *SLT1* to *SLT5*, associated with spontaneous incidence of lung tumors [7]. Our analysis in 27 strains contained 12, 4, 10, 11, and 19 SNPs in the *SLT1* to *SLT5* regions, respectively. However, none of the *SLT* loci were confirmed in our GWA analysis (Table 3). To rule out the possibility that the non-replication of previous results [7] was due to the lack of inclusion of relevant SNPs in the SNP database used, we identified and selected SNPs in the same *SLT* regions showing exactly the same 13 strain distribution pattern reported [7] and genotyped the selected SNPs in the 27 strains of our study (Table 1) plus the O20/A strain [5]. However, none of the SNPs located in *SLT1* (rs13478866), *SLT2* (rs13479117), *SLT3* (*Galnt2* JC10664_20, *Agt* JC10667_5, *Agt* JC10669_3), *SLT4* (rs13483600), and *SLT5* (rs3667513) confirmed an association with spontaneous lung tumor incidence (not shown) and none of the *SLT* loci showed an association with urethane-induced lung tumor incidence or multiplicity (Table 3).

We also tested whether previous GWA results on lung tumor multiplicity [8] might be confirmed in our study. Unlike the spontaneous incidence data, the sizes of the two datasets on lung tumor multiplicity are almost identical (n = 21–22) [8], with very similar strain composition (Table 1 and [8]). Differences included two BALB/c substrains and the O20/A strain analyzed in [8], whereas we analyzed only one BALB/c substrain and did not include the O20/A strain but did include the C58/J and the NZB/BlGd strains not analyzed in [8]. Overall, we expected to observe essentially overlapping results. Using the WTCHG 13K SNP panel, we confirmed the association at the *Pas1* locus, with 2 SNPs showing P values above the statistical threshold for the tumor incidence phenotype (Table 3). At the *Clas2* locus on Chromosome 4, (CEL-4_30653207, alias rs27801920), [8], we found a −log P = 2.8. Genotyping at this locus in the strains of the genome-wide scan plus the NGP/N and O20/A strains for the functional *Lasc1* SNP D102E (rs32396036) revealed no significant associations with lung tumor phenotypes (−log P = 1.8 to 2.4). Furthermore, we detected no significant associations at the *Clas3* and *Clas4* regions (Table 3), despite the inclusion of 6 and 9 SNPs in the *Clas3* and *Clas4* regions, respectively. Even the higher SNP density offered by the BROAD SNP panel failed to reveal any *Clas* loci except *Pas1* (*Clas1*).

Another recent GWA study conducted in 20 inbred strains treated with N-nitroso-N-ethylurea and scanned with the WTCHG SNP database detected several putative lung tumor susceptibility loci on Chromosomes 3, 6, 9, and 15 [9]; these loci did not correspond to *Clas* loci or to regions detected in the present study. Authors of [9] did not detect the *Pas1* locus, which has been implicated in lung tumorigenesis independently of the type of chemical carcinogen [5]. In contrast with the GWA results, the *Pas1* locus but none of the GWA loci was detected in a genetic linkage study that used the same carcinogen as in the GWA study, i.e., N-nitroso-N-ethylurea [10]. We observed no association with any lung tumor phenotype at any of the loci reported in [9], using either the WTCHG or the BROAD SNP panel.

### Loci Detected by Strain Survey Are Not Confirmed by Genetic Linkage Studies

Genetic linkage analysis of mouse crosses represents a formal approach to demonstrating functional activity of genetic loci on given phenotypes. In a single cross, not all loci affecting a specific complex phenotype in a given species are expected to be detected, since a locus can exert allele-specific effects only in crosses originating from two strains carrying different alleles and cannot be detected if the functional element(s) is non-polymorphic in the two parental strains. Accordingly, the *Pas1* locus is easily detected in crosses between strains carrying either of the two *Pas1* alleles (or haplotypes) but is not detectable in crosses between strains carrying the same haplotype [11],[12].

**Figure 3:**
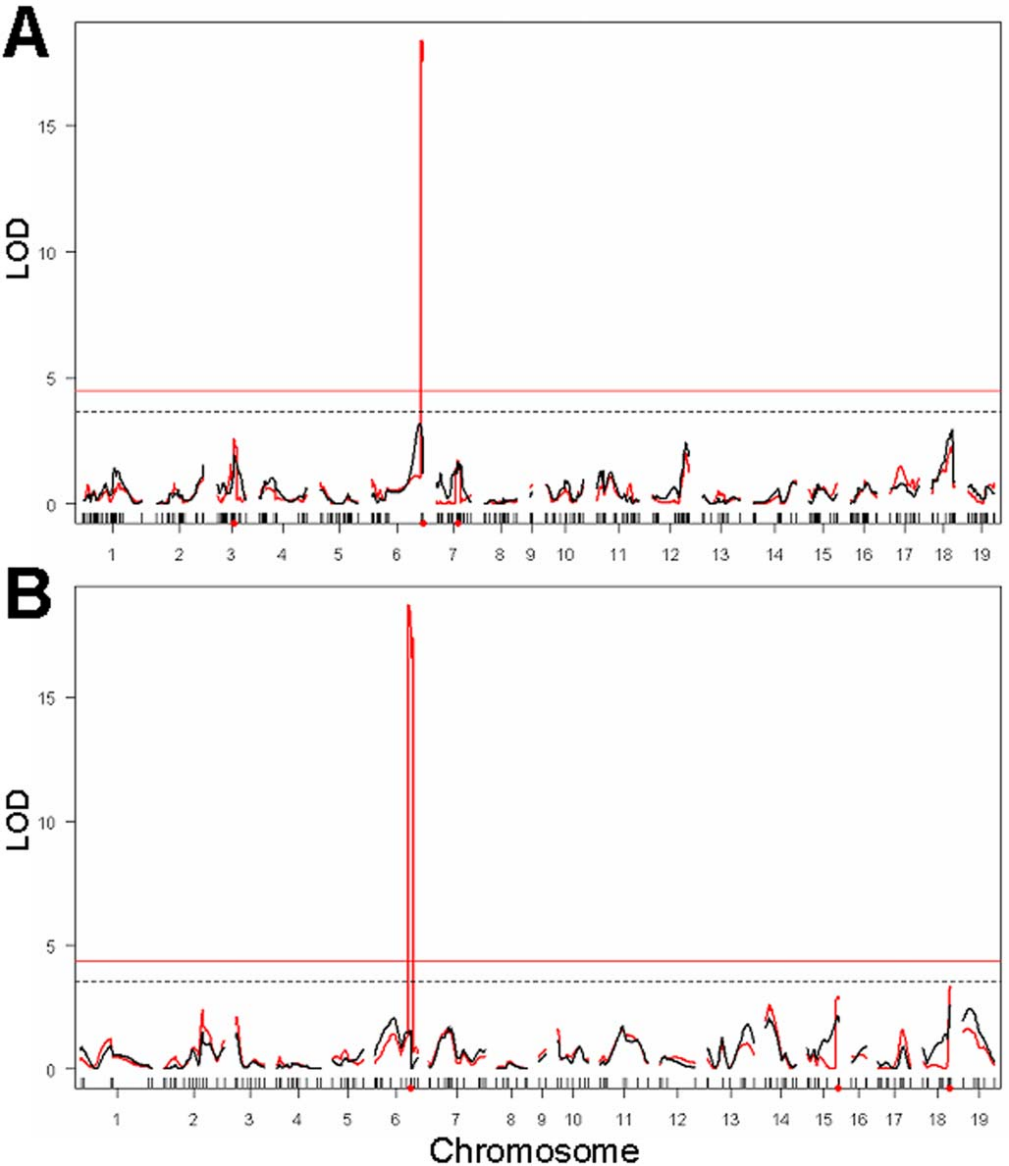
Genome-wide genetic linkage analysis of loci affecting urethane-induced lung tumor multiplicity. (A) (BALB/c×C3H/He)F2 cross detects the *Pas1* locus at LOD score = 18.4. (B) (A/J×C3H/He)F2 cross detected the *Pas1* locus at LOD score = 18.7. Red curves indicate the results of the composite interval mapping, whereas black curves indicate the results of genome scan using the *Kras* genotype as covariate (conditioning on the *Pas1* alleles). Horizontal lines indicate the threshold values (α = 0.05) of the LOD score. The *Clas2* locus (Chromosome 4) showed no significant linkage, despite the presence of the claimed functional polymorphism (D102E) in both crosses. No other locus detected by whole-genome strain survey showed significant linkage.

To test whether loci detected by genome-wide strain survey may be involved in modulating lung tumorigenesis, we carried out genome-wide genetic linkage analyses of three intercross populations previously analyzed for urethane-induced lung tumor multiplicity [11]–[13]. In our population of (BALB/c×C3H/He)F2 mice, genotyping by SNP array detected a total of 383 non-redundant informative SNPs widely dispersed over the whole mouse genome. There was complete coverage of all chromosomes, with a range of 12–43 non-redundant SNPs genotyped for each chromosome, except for Chromosome 9 which contained only 4 SNPs. Above the R/qtl threshold, only the effect of the *Pas1* locus was observed, with LOD scores of 18.4 (Figure 3A, red line). By conditioning on the *Pas1* genotype, no additional QTLs were detected (Figure 3A, black line). In (A/J×C3H/He)F2 mice, 192 markers ensured genome-wide coverage, and composite interval mapping scan detected the known *Pas1* locus and no other locus (Figure 3B, red line), even by a separate conditioning for the *Pas1* genotype (Figure 3B, black line). Analysis of the (BALB/c×SWR/J)F2 population by composite interval mapping scan confirmed the reported Chromosomes 4 (*Papg1*), 6 (*Par4*), and 18 (*Par2*) loci and detected an additional locus (LOD score = 5.3) on Chromosome 1 between *D1Mit18* and *D1Mit22* markers (not shown).

Thus, none of the loci except *Pas1* identified by previous or the present (Table 3) genome-wide strain survey were confirmed by genetic linkage analysis (Figure 3), although either the same or flanking SNPs detected by GWA at *SLT1* to *SLT6* loci and at *Clas1* (*Pas1*) to *Clas5* loci were, in fact, polymorphic in at least one of our three intercross populations.

Note that the *Clas2* locus showed no effect in the (BALB/c×C3H/He)F2 cross, as indicated by the absence of any significant linkage of the whole Chromosome 4 (covered by 19 SNPs) to any lung tumor phenotype (Figure 3 and not shown). Moreover, since the claimed functional D102E polymorphism of the *Lasc1* gene defining the *Clas2* locus [8] was present in the (BALB/c×C3H/He)F2 cross, we genotyped that polymorphism; no significant linkage with lung tumor multiplicity was found at α = 0.10 significance level.

Further testing of the D102E polymorphism by genotyping in the (A/J×C3H/He)F2 cross (total of 163 mice) [13], which also carries the polymorphism, again revealed no significant associations with either lung tumor multiplicity or volume (Figure 3B).

With regard to the loci detected by haplotype analysis, none but those linked with the *Pas1* locus were confirmed by genetic linkage analysis. Except for the *Halt2* locus on Chromosome 14, which could not be detected since all four parental strains of our genetic crosses carried the same haplotype, all other *Halt* loci could be detected in at least one of the intercrosses that displayed informative haplotypes (Table 4). In the (BALB/c×SWR/J)F2 intercross, a significant linkage was found in the pulmonary adenoma resistance 2 (*Par2*) locus [12], with peak LOD score = 15.5 at *D18Mit33*, located at about 10 Mb distal from the *Halt7* locus; no linkage near the *Halt7* locus was found in the two other informative crosses (Table 4).

### Absence of *Lasc1* mRNA Expression in Mouse Lung

**Figure 4:**
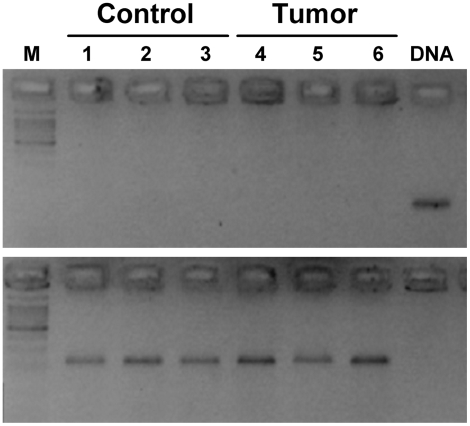
Absence of *Lasc1* gene expression in mouse normal lung and lung tumors of (A/J×C57BL/6J)F1 mice. The ethidium bromide-stained gel shows the RT-PCR results: lanes 1–3, normal lung derived from adult mice; lanes 4–6, lung tumors derived from urethane-treated mice; lane M, DNA size marker; lane DNA, genomic DNA. Only genomic DNA (top panel) and the *Itpr2* housekeeping gene (bottom panel) were amplified.

Notwithstanding the lack of significant linkage of the *Lasc1* gene in two independent crosses, we examined *Lasc1* mRNA expression in mouse normal lung and lung tumors. RT-PCR analysis of normal lung tissue from A/J and C57BL/6J mice, which carry different alleles of this gene, and of normal lung and tumor tissue from (A/J×C57BL/6J)F1 mice revealed no *Lasc1*-specific transcript fragments in either normal or tumor lung tissue, whereas genomic DNA was clearly amplified (Figure 4, top) and the *Itpr2* or *Gapdh* positive controls were readily detected in cDNA samples (Figure 4, bottom and data not shown). In light of the reported widespread expression of *Lasc1* [8], we examined *Lasc1* mRNA expression in brain, liver, kidney, spleen, and testis of adult mice; only testis from which the AK076999 clone was originally derived revealed detectable *Lasc1* mRNA (not shown).

## Discussion

We found a highly significant correlation (r = 0.94) between the phenotypes of spontaneous incidence and carcinogen-induced multiplicity of mouse lung tumors (Figure 1). Although we cannot exclude the possibility that part of this correlation rests in population structure, the result suggests that the genetic control of both phenotypes resides mainly in the same genetic loci. The high correlation between the two phenotypes may be explained by the *Pas1* locus and its strong effects on both phenotypes, i.e., odds ratios of ∼12 and ∼15 with spontaneous and chemically induced lung tumorigenesis, respectively [5]. At present, it is not known whether additional lung tumor modifier loci can control both phenotypes. Proof that the same genetic elements control both spontaneous and chemically induced lung tumorigenesis in the mouse model could have important implications for other species, including humans, and therefore warrants further study.

The design of the present GWA study was similar to that of two previous studies carried out for either spontaneous [7] or urethane-induced incidence of lung tumors [8], although we had the opportunity to analyze a larger number of mouse strains. Indeed, in the spontaneous tumorigenesis association, we analyzed 27 strains with 13K SNPs (WTCHG) and 23 strains with 140K SNPs (BROAD) versus 13 strains with ∼135,900 SNPs [7]. In the analysis of urethane-induced lung tumorigenesis, we analyzed 20–22 (WTCHG and BROAD) strains for tumor multiplicity and incidence (Table 1), respectively, versus an effective number of 19 strains with ∼123,000 SNPs in [8], where the two BALB/c substrains should count as a single strain because of their overlapping phenotypes and genotypes, and where lack of available genotype data from the Broad Institute for the C57BL/10J strain allowed analysis only with the 13K WTCHG panel of SNPs.

The power to detect genotype-phenotype associations depends on the genomic length over which LD between functional and marker polymorphisms extends. Since LD decays with distance, a high-density map provides better resolution power than a low-density map. However, the haplotype structure of mouse inbred strains shows a mosaic pattern [14], and haplotype segments ranging from 12 to 608 kb in length have been reported [15]. Those findings suggest that even a medium-density map is sufficient to detect QTLs, especially considering the limited pool of founder genomes of the mouse laboratory strains and their consequent relatedness [16]. Indeed, our use of the BROAD high-density SNP panel (∼20 kb per SNP) confirmed the *Pas1* detection but not the *SLT6* and *Clas5* loci detected by the medium-density WTCHG SNP panel (average density of ∼160 kb per SNP). On the other hand, the power of QTL detection decreases as strain number decreases, and it has been proposed that there is little rationale supporting analysis of complex traits using less than 30 strains [17]. For comparisons, population-based association studies in humans require several hundreds or thousands of individuals, and confirmation of the results is also required [18].

We confirmed none of the 5 *SLT* loci detected in previous GWA studies. The previous association study on the urethane-induced lung tumor incidence phenotype detected 4 loci (*Clas1* to *Clas4*) [8], none of which overlap with any of the *SLT* loci identified by the same group in another study [7]. Using the same phenotype, we confirmed the *Pas1* locus (also called *Clas1* in [8]), with 2 SNPs in both the WTCHG (rs13459098 in the *Casc1* gene and rs13479086 in the genomic region between *Kras* and *Ifltd1* genes) and the BROAD panel (rs30118733 at 5′-end of the *Pas1* haplotype and rs30752783 near the *Ifltd1* gene) showing statistical associations. However, the *Clas2* to *Clas4* loci were not confirmed; genotyping of the functional *Clas2* element (D102E, rs32396036) in all strains for which phenotype data were available revealed a −log P = 2.4 for urethane-induced lung tumor incidence and a lower statistical association for the other tumor phenotypes (Table 3).

The discrepancy regarding *Clas2* to *Clas4* detection in our study and that of [8] may rest in small differences in strain composition. The reported functional D102E polymorphism at the *Lasc1* gene (*Clas2* locus) may represent either a locus with a very weak effect or a false-positive finding, since no significant linkage was detectable in either (BALB/c×C3H/He)F2 or (A/J×C3H/He)F2 intercrosses carrying that polymorphism, and no *Lasc1* transcript was detectable in normal lung tissue, in lung tumors, or in several mouse organs, except testis, despite its reported widespread expression [8]. Thus, the reported allele-specific effects by *in vitro*-transfected expression vectors containing either the 102D or 102E *Lasc1* allele (whose cDNA is contained in a single exon; Vega gene OTTMUSG00000004898, http://vega.sanger.ac.uk/index.html) cannot constitute evidence of a locus effect in the absence of such evidence by genetic linkage studies.

Haplotype analysis did not increase the reliability of GWA in comparison with single-point analysis, since the *Halt* loci detected by haplotype analysis, with the exception of those linked to *Pas1*, were not confirmed by genetic linkage studies despite the haplotype differences between the parental strains originating the crosses. The *Halt7* locus might also represent a false-positive association, since significant genetic linkage near the *Halt7* locus position was detected in only one of three informative intercrosses, and since the mapping position of *Halt7* is ∼10 Mb apart from the LOD score peak defining the *Par2* locus (69.83 Mb) [12] and its candidate gene *Poli* (70.67 Mb) [19].

Overall, our comparison of the results of GWA studies in inbred strains with the genetic linkage analysis results confirmed none of the putative loci identified by strain survey, except the *Pas1* locus (Figure 3), notwithstanding the polymorphism of several loci in the genetic cross examined. Comparison of our GWA results with those of previous studies indicates a high variability of the statistical thresholds obtained by permutation. This is expected, since the thresholds are influenced by the number of SNPs and of strains, by the correlation structure of the data, and by the distribution of the phenotypes under study. Thus, inclusion or exclusion of even a single strain in a 20-strain study would strongly affect the statistical thresholds and the loci detected. Our study raises concern about the ability of GWA studies to detect authentic QTL loci and provides a note of caution to the mouse genetics field, where GWAs are seeing wide application across many phenotypes. In agreement with previous studies [17],[20], our results support the notion that association mapping in the population of inbred mouse strains is characterized by a high false-positive rate and that such a method must be carried out with a large number of strains (i.e., 40 to 150). Accordingly, extensive computer simulation analyses have shown that the power of GWAs studies is low for phenotypes controlled by polygenic traits and that spurious associations are expected [21]. Thus, GWA studies should be carried out in conjunction with genetic linkage analysis to detect relevant loci.

## Materials and Methods

### Mouse Phenotypes, DNAs, and RNAs

Table 1 lists the data for 32 mouse inbred strains on spontaneous lung tumor incidence (n = 28), urethane-induced lung tumor multiplicity, i.e., number of tumors/mouse (n = 24), and urethane-induced lung tumor incidence (n = 21) derived from [5],[7],[22],[23]. Genomic DNAs from the same inbred strains were obtained from The Jackson Laboratory Mouse DNA Resource (Bar Harbor, ME, USA). Intercross populations consisted of (BALB/cJ×C3H/HeJ)F2 mice (n = 182 males) [11], (A/J×C3H/He)F2 (n = 87 males and 87 females) [13], and (BALB/c×SWR/J)F2 mice (n = 106 males and 112 females) [12]; all three populations had been treated with a single dose of urethane, observed without any further treatment, and evaluated quantitatively for lung tumor multiplicity phenotype. RNA was extracted from normal lung of adult male A/J, C57BL/6J, and (A/J×C57BL/6J)F1 mice, from urethane-induced lung tumors of (A/J×C57BL/6J)F1 mice [4], and from brain, liver, kidney, spleen, and testis of a male SM/J adult mouse, using the NucleoSpin RNA II kit (Macherey-Nagel, Bethlehem, PA, USA).

### SNP Genotype Extraction, Genome-Wide Scan, and SNP Genotyping

Genotypes of 12,959 and 138,793 SNPs publicly available at Wellcome Trust Centre for Human Genetics (WTCHG) (http://www.well.ox.ac.uk/mouse/INBREDS/) and at Broad Institute (http://www.broad.mit.edu/), respectively, were extracted. Table 1 lists the strains for which WTCHG or BROAD genotypes are available. Genomic DNAs of (BALB/cJ×C3H/HeJ)F2 mice were genotyped using Illumina SNP genotyping technology which allows the simultaneous analysis of 1536 SNPs [24]. Genomic DNAs of (A/J×C3H/He)F2 mice were genotyped using MassARRAY (Sequenom, Inc., San Diego, CA) with a multiplex PCR assays (iPLEX) designed by Sequenom SpectroDESIGNER software. The extension products were spotted onto a 384-well spectroCHIP before analysis by MALDI-TOF mass spectrometry. Selected SNPs were genotyped in mouse inbred strains by pyrosequencing on a PSQ96MA system (Biotage AB, Uppsala, Sweden). A short fragment containing the SNP was PCR-amplified using a biotinylated primer as one of the two PCR primers and pyrosequenced according to the manufacturer's instructions.

### *Lasc1* Expression Analysis

*Lasc1* mRNA was searched by RT-PCR using primers: 5′-tactcactggtggtcctaagatcg-3′ and 5′-aggaaaaatggcccttccg-3′; which flank the reported D102E polymorphism of the AK076999 cDNA sequence and, according to the *Lasc1* gene structure (Vega gene OTTMUSG00000004898, http://vega.sanger.ac.uk/index.html), are located in the same exon. The *Itpr2* (5′-tgatggacaccaagctgaag-3′ and 5′-cgaacattgtttctgcctga-3′) and *Gapdh* (5′-tgttcctacccccaatgtgt-3′ and 5′-gtggaagagtgggagttgct-3′) genes served as positive controls. Normal lung tissue from 3 A/J, 3 C57BL/6J, and 3 (A/J×C57BL/6J)F1 mice and lung tumors from 3 urethane-treated (A/J×C57BL/6J)F1 mice were used, as well as brain, liver, kidney, spleen, and testis of a male SM/J mouse.

### Statistical Analysis

The association between spontaneous and urethane-induced lung tumor phenotypes (mean percentages or mean multiplicities) was expressed as a correlation coefficient. The association between each SNP and lung tumor phenotypes (expressed as log+1 of phenotype value) was tested by t-test. Haplotype analysis was carried out according to [25], using a sliding window approach (window size of 3 SNPs) and the BROAD dataset. The association between each haplotype and lung tumor phenotypes (expressed as log+1 of phenotype value) was tested by F-test. To control the genome-wide false-positive fraction (12,959 and 138,793 t-tests for databases WTCHG and BROAD), statistical thresholds were computed both in accordance with the Bonferroni principle and with a permutation test (20,000 permutations). In particular, the distribution of the 20,000 smallest p-values among the 12,959 (or 138,793) p-values under the null hypothesis was obtained. This approach implicitly uses the correlation structure of the data [26]. The 5^th^ and 10^th^ centile of the reference distribution of the smallest p-values were used to guarantee a 0.05 or 0.10 overall false-positive fraction.

Genome-wide genetic linkage was carried out by interval mapping using R/qtl [27]. Marker order and position on chromosomes were established by multipoint analysis of the data using the MAPMAKER/EXP program [28]. Genetic distances were computed using Haldane's mapping function. Single-locus genome scans were carried out using the ‘scanone’ function of R/qtl (http://www.biostat.jhsph.edu/˜kbroman/qtl/) using the Haley-Knott regression analysis. To increase the power to detect weak QTLs and to condition on the presence of the *Pas1* genotype, a composite interval mapping was carried out using the three most significant markers identified by a stepwise regression as covariates [29]. In addition, the *Kras2* genotype was used as a covariate in a single-locus genome scan of (A/J×C3H/He)F2 and (BALB/c×C3H/He)F2 intercross populations. Genome-wide significance thresholds (α = 0.05) were generated through permutation tests (10,000 permutations) as described [30].
